# *Spilanthes filicaulis* (Schumach. & Thonn.) C.D. Adams leaves protects against streptozotocin-induced diabetic nephropathy

**DOI:** 10.1371/journal.pone.0301992

**Published:** 2024-04-19

**Authors:** Oluwafemi Adeleke Ojo, Akingbolabo Daniel Ogunlakin, Christopher Oloruntoba Akintayo, Olaoluwa Sesan Olukiran, Juliana Bunmi Adetunji, Omolola Adenike Ajayi-Odoko, Theophilus Oghenenyoreme Ogwa, Olorunfemi Raphael Molehin, Omolara Olajumoke Ojo, Ramzi A. Mothana, Abdullah R. Alanzi

**Affiliations:** 1 Phytomedicine, Molecular Toxicology, and Computational Biochemistry Research Laboratory (PMTCB-RL), Department of Biochemistry, Bowen University, Iwo, Nigeria; 2 Department of Physiology, Afe Babalola University, Ado-Ekiti, Nigeria; 3 Department of Physiology, Obafemi Awolowo University, Ile-Ife, Nigeria; 4 Department of Biochemistry, Osun State University, Osogbo, Nigeria; 5 Department of Microbiology, Bowen University, Iwo, Nigeria; 6 Department of Biochemistry, Ekiti State University, Ado-Ekiti, Nigeria; 7 Department of Pharmacognosy, College of Pharmacy, King Saud University, Riyadh, Saudi, Arabia; Jadavpur University, INDIA

## Abstract

**Background and objective:**

Diabetic neuropathy (DN) is a complex type of diabetes. The underlying cause of diabetic nephropathy remains unclear and may be due to a variety of pathological conditions resulting in kidney failure. This study examines the protective effect of the methanolic extract of *Spilanthes filicaulis* leaves (MESFL) in fructose-fed streptozotocin (STZ)-induced diabetic nephropathy and the associated pathway.

**Methods:**

Twenty-five rats were equally divided randomly into five categories: Control (C), diabetic control, diabetic + metformin (100 mg/kg), diabetic + MESFL 150 mg/kg bw, and diabetic + MESFL 300 mg/kg bw. After 15 days, the rats were evaluated for fasting blood glucose (FBG), alanine aminotransferase (ALT), aspartate aminotransferase (AST), alkaline phosphatase (ALP), urea, uric acid, serum creatinine, reduced glutathione (GSH), superoxide dismutase (SOD), catalase (CAT), and lipid peroxidation (MDA). Gene expression levels of cyclic adenosine monophosphate (cAMP), protein kinase A (PKA), cAMP response element-binding (CREB), cFOS and the antiapoptotic protein Bcl-2 were examined.

**Results:**

We observed that MESFL at 150 and 300 mg/kg bw significantly downregulated the protein expression of cAMP, PKA, CREB, and cFOS and upregulated the Bcl-2 gene, suggesting that the nephroprotective action of MESFL is due to the suppression of the cAMP/PKA/CREB/cFOS signaling pathway. In addition, MESFL increases SOD and CAT activities and GSH levels, reduces MDA levels, and reduces renal functional indices (ALP, urea, uric acid, and creatinine).

**Conclusion:**

Therefore, our results indicate that MESFL alleviates the development of diabetic nephropathy via suppression of the cAMP/PKA/CREB/cFOS pathways.

## Introduction

Diabetes mellitus (DM) is a chronic condition that is characterized by a lack of insulin, high blood sugar levels, dyslipidemia, and impaired nephrotic function. Previous studies have shown that prolonged high blood sugar levels can lead to irreparable damage to several important human organs, including the eyes, kidneys, and heart [1]. Diabetes-related nephropathies (DN) are one of the leading complications associated with diabetes. The incidence of DN is rising rapidly in tandem with the diabetes epidemic [2]. Approximately 25 to 50% of people with diabetes develop renal dysfunctions [3]. Diabetic nephropathy has been linked to increased renal dysfunction and mortality [4]. For this reason, the primary objective of DN treatment remains to treat hyperglycemia [5]. Oral hypoglycemic agents used in DM management therapies must improve pathophysiology and inhibit glucose production in the liver. Furthermore, the hypoglycemic agents used to treat DN are adipogenic agents [6]. Synthetic oral hypoglycemic agents are highly toxic [7].

Dysregulation of biochemical pathways occurs due to increased blood glucose levels, accumulation of reactive oxygen species (ROS) and excessive production of reactive oxygen species that cause tissue injury [8, 9]. Factors that contribute to DN progression but are not limited to include hyperglycemia, ketoacidosis, hypertension, and obesity. However, the precise mechanism remains unclear. Oxidative stress plays a role in the development of diabetic nephropathy [10]. In addition, ROS play a critical role in DN pathogenesis. However, the mechanisms underlying several abnormal physiological activities in DN pathogenesis are complicated. It is crucial to understand these pathways to identify novel therapeutic approaches to enhance patient outcomes.

*Spilanthes filicaulis* (Schumach. & Thonn.) C.D.Adams syn. (*Acmella caulirhiza* Delile) is a shrubby plant of the family Asteraceae that is often discovered in tropical and subtropical areas, including India, Malaysia, America, Africa, and West Africa [11]. *Spilanthes filicaulis* spp. is applied in the following ways: snakebite treatment (Ghana) using the aerial part of the plant [12], emetic treatment using its leaves [13], intestinal diseases and diarrhea treatment, chest pain, eczema, guinea worm, and toothache treatment using the whole plant [14]. Although, evidence of its usage in folkloric medicine for managing diabetes exits, few studies have reported the antidiabetic potential of *Spilanthes filicaulis*. Recently, Ojo et al. [15] reported the antioxidant, anti-diabetic, and anti-inflammatory activities of the ethyl acetate fraction of *S*. *filicaulis* leaf. Furthermore, *S*. *filicaulis* aqueous extract had complete mucosal cytoprotection in HCl/EtOH-induced gastric lesions in male Wistar rats [16]. Ethanol extract of *Spilanthes filicaulis* leaf also attenuated abnormal on liver biochemical indices inn carbon tetrachloride induced hepatic damage in Wistar rats [17].

Other pharmacological activities of *Spilanthes filicaulis* investigated on animal models include antidiarrheal, antimicrobial, anthelminthic, antioxidant, etc. [18–20]. Currently, it is thought that the genus *Spilanthes* spp. is active due to five constituent groups: alkamides, coumarins, flavonoids, terpenoids, and polysaccharides. In general, extracts from *Spilanthes* spp. are employed more often than isolated substances in the pharmaceutical, medical, traditional, and cosmetic industries. This suggest why *Spilanthes* spp. extract performs better than isolated molecules. Given the quantity of chemicals present in an extract from a medicinal plant, it appears highly probable that pharmacokinetic potentiation or pharmacodynamic augmentation will occur. The bioavailability of the biomolecules that reach the target sites to provide the necessary pharmacological impact is what determines whether the presence of plant secondary metabolites in an extract is adequate to exert a pharmacological effect. Ojo et al. [21] reported the presence of 13-octadecenal, ar-tumerone, tetrasoxane, oxirane, and oleic acid, 4-hydroxy-3-methylacetophenone, 2-methyl-Z,Z-3,13-octadecadienol, 1-monolinoleoylglycerol trimethylsilyl ether, cis-13-octadecenoic acid, and 3,11-tetradecadien-1-ol in *Spilanthes filicaulis* Leaf methanol extract, via GC-MS studies. Among these compounds, alpha-caryophyllene and piperine had the potential of inhibiting dipeptidyl peptidase-IV (DPP-IV) activity [21]. In addition, Ilondu et al. [22] reported the presence of listed phytochemicals, including flavonoids, phenols, glycosides, alkaloids, steroid glycosides and terpenes, in ethanolic extracts of *Spilanthes filicualis* leaves. Furthermore, the antidiarrheal activity of *S*. *filicaulis* leaf decoction used in traditional medicine and the antibacterial and anti-adhesive properties of these plants may be due to the presence of flavonoids [18, 20, 23–26].

Cyclic adenosine monophosphate (cAMP) is a type of nucleotide that is formed when two phosphoric acids are condensed and excreted by ATP. This nucleotide is a key secondary messenger in numerous feedback mechanisms and in the transduction of cell signals. It is the main activating factor of protein kinases A (PKA) and exchange protein (Epac) [27, 28]. Furthermore, PKA activates cAMP-responsive element-binding protein (CREB), which serves as a regulator to control gene transcription, including the production of Cyclin D1 and Cyclin C-Jun [29]. Thus, the cAMP/PKA/CREB/cFOS pathway is a common intracellular pathway that performs an essential function in the development of DM [30, 31]. This suggests that inhibiting this pathway could be a viable approach to reduce damage to kidney cells in DN pathological conditions. Hence, there is need to examine the influence of botanicals on cAMP/PKA/CREB/cFOS signaling among diabetics, with the aim of obtaining evidence that could be used to elucidate the pathophysiology of DN. Therefore, this study examined the effect of *Spilanthes filicaulis* leaves on streptozotocin-induced diabetic nephropathy and its influence on cAMP/PKA/CREB/cFOS signaling.

## Materials and methods

### Plant material

*Spilanthes filicaulis* plants were obtained from a local farm settlement in Iwo-Oluponna (7° 35′ 34.7°N 4° 11′ 27.5°E), Osun state, and the leaves were manually obtained. Authentication and identification of the plant was conducted at the Department of Pure and Applied Biology, Bowen University, Iwo Osun state with herbarium number BUH035.

### Chemicals

All chemicals and reagents used were of analytical grade, and the Streptozotocin used was procured from Glentham Life Sciences Ltd., Corsham, United Kingdom.

### Experimental animals

Twenty-five healthy male Wistar rats weighing 140–170 g were procured for the purpose of this study. The rats were housed in decontamination cages under specific conditions and were acclimatized for one week, with access to rat feed (pellet) and uncontaminated water. In compliance with the criteria and guidelines indicated in the National Institute of Health (NIH) guidelines for the care and usage of laboratory animals, an ethical approval number was provided (BUAC/BCH/2023/0002B).

### *Spilanthes filicaulis* methanolic extract preparation

The manually obtained leaves were air-dried and mashed. The extraction of the sample was obtained by immersion of a known mass of the leaf powder (34.7 g) in 70% methanol (300 ml) in an airtight beaker, which was left to stand for 72 hours. Filtration of the extract was performed using a muslin cloth, with further concentration using a water bath and oven at 70°C [15].

#### Animal grouping

The animals were categorized into five groups, with five animals in each category. Group allocation is indicated below.

Group A: Normal control (nondiabetic, received feed and distilled water daily).
Group B: diabetic control (untreated, received just feed and water daily).
Group C: Diabetic + Metformin (100 mg/kg body weight)
Group D: Diabetic + *S*. *filicaulis* methanolic extract (low dose of 150 mg/kg).
Group E: Diabetic + *S*. *filicaulis* methanolic extract (high dose of 300 mg/kg).

#### Induction of type-2 diabetes

The diabetic animals in groups B, C, D, and E were induced with diabetes intraperitoneally using STZ (STZ) at a low dose of 40 mg/kg body weight prepared in citrate buffer at pH 4.5 after the administration of fructose for two weeks prior to induction. The induction was performed after the animals were fasted for 12 hours; thereafter, the fasting blood glucose level was determined using a glucometer. Blood samples were obtained from the tail end of the rat using a lancet, and animals with fasting blood glucose levels greater than 250 mg/dl after 48 hours were considered diabetic. The experiment was conducted for a duration of 14 days [32]. The rat care procedures were approved by the Department of Biochemistry, Bowen University animal ethics committee, and the study was authorized with the identification number BUAC/BCH/2023/0002B.

#### Animal sacrifice and sample collection

Animal euthanasia was performed by placing the animals under anesthesia using 3 ml of ketamine, which was administered intramuscularly. An incision was made on the abdominal cavity, and the skin was reflected to expose the abdominal and thoracic region. Blood samples were obtained in plain samples from the right ventricle of the heart using a syringe and were left to stand at room temperature undisturbed for approximately 30 minutes before centrifugation at 3000 r.p.m. for 5 minutes. The obtained serum was labeled appropriately for further biochemical analysis. The liver was procured carefully and weighed, after which homogenization was performed using ice cold phosphate buffer in a mortar and pestle. Centrifugation of the homogenates was performed at 5000 r.p.m for 10 minutes, after which the supernatant was obtained and stored in the refrigerator for further biochemical analysis.

### Determination of biochemical parameters

The standard procedures adopted were as described for fasting blood glucose [33], catalase [34], superoxide dismutase (SOD), reduced glutathione (GSH) [35], malondialdehyde (MDA) [36], alkaline phosphatase (ALP) [37], aspartate aminotransferase (AST), and alanine aminotransferase (ALT) [38]. Other procedures adopted were as described for creatinine [39], urea [39], and uric acid [40]. Serum insulin concentration determination was achieved based on the method described by [32], which used an ELISA kit from Sweden in a multiple plate ELISA reader (Winooski, Vermont, USA). The homeostasis model assessment of insulin resistance (HOMA-IR) and homeostasis model assessment of the β-cell score (HOMA-β) were calculated through the Eqs (1) and (2) described by [32].


$$HOMA-IR=\frac{[insulin\;\left(U/L\right)X\;blood\;glucose\;\left(mmol/L\right)}{22.5}$$ (1)

$$HOMA-\beta =\frac{\left[20\;X\;insulin\;\left(U/L\right)\right]}{\left[blood\;glucose\;\left(mmol/L\right)-3.5\right]}$$ (2)

Converting factors for units: insulin (1 U/L = 7.174 pmol/L) and blood glucose (1 mmol/L = 18 mg/dL).

### Gene expression study

#### Isolation of total RNA

Total RNA was extracted from kidney tissues using the Quick-RNA miniPrep^™^ Kit from Zymo Research. DNA contamination was eliminated after DNAse I treatment (NEB, Cat: M0303S). The total RNA concentration was measured at 260 nm. The purity of the RNA was determined at 260 nm and 280 nm by an A&E spectrophotometer (A&E Lab, UK).

### cDNA conversion

Reverse transcriptase reaction was used to transform one (1) microgram (DNA-free RNA) into cDNA with the use of a cDNA synthesizing kit using ProtoScript II First-Strand technology (New England BioLabs). The reaction temperature was set to three different conditions: 65°C for 5 min, 42°C for 1 h, and 80°C for 5 mins [33, 41].

### PCR amplification and agarose gel electrophoresis

A polymerase chain reaction was used to amplify the gene of interest using NEB (OneTaqR 2X Master Mix) with the following primers (Inqaba Biotec, Hatfield, South Africa). PCR amplification was performed in a 25 μl reaction mixture containing cDNA, primers (forward and reverse SEE BELOW Table 1) and Ready Mix Taq PCR master mix. Under the following conditions: initial denaturation at 95°C for 5 min, followed by 30 cycles of amplification (denaturation at 95°C for 30 s, annealing for 30 s and extension at 72°C for 60 s) and a final extension at 72°C for 10 min. The amplicons were resolved on a 1.0% agarose gel. The GAPDH gene was used to normalize the relative level of expression of each gene, and quantification of band intensity was performed using ImageJ software [33, 42].

**Table 1:** Primer sequences.

Gene	Forward	Reverse
cAMP	5’- TACTCCGTGCTGTGGATGACTT -3’	5’- TCTTGAACCGGAAAGGCTGTAT -3’
PKA	5’- CCGAACTTGGACCTTGTGTGG -3’	5’- CGCACCTTCCCAGAGACGATT-3’
CREB	5’- CATTGCCCCTGGAGTTGTTAT-3’	5’- CTCTTGCTGCTTCCCTGTTCTT-3’
cFOS	5’- CAGCCTTTCCTACTACCATTCCC-3’	5’- CAGGAGATAGCTGCTCTACTTTGC-3’
Bcl2	5’- GCGTCAACAGGGAGATGTCA-3’	5’- TTCCACAAAGGCATCCCAGC-3’
GAPDH	5’-CTGGAGAAACCTGCCAAGTATG-3’	5’- GGTGGAAGAATGGGAGTTGCT-3’

### Histopathological examination of the kidney tissues

A standard laboratory protocol for paraffin embedding was used to treat the formalin-preserved kidney tissues of the male rats. Tissue sections (4 mm) were fixed to slides, deparaffinized in *p*-xylene, rehydrated in ethanol (100, 80, 70, and 50%) and rinsed with water. Slides were stained for 5 min in hematoxylin, rinsed with water, counterstained in eosin, mounted in dibutylphthalate polystyrene xylene, cover-slipped, and viewed at x 100 with a Leica slide scanner (SCN 4000, Leica Biosystems, Wetzlar, Germany).

### Statistical analysis

The results are expressed as the mean ± SEM (n = 5). The means were analyzed using a one-way analysis of variance followed by a Duncan Multiple Range *post hoc* Test (DMRT) at *p* < 0.05 using GraphPad version 8.0 (GraphPad Software, Inc., San Diego, California, USA).

## Results

### Effects of the methanolic extract of *Spilanthes filicaulis* leaves on the biochemical parameters of STZ-induced diabetic rats

#### Fasting blood glucose

The fasting blood glucose levels of the male Wistar rats used in this study were elevated 72 hours (3 days) after the induction of diabetes with streptozotocin with values up to 400 mg/dL. Throughout the duration of the experiment (15 days), the untreated group maintained these elevated blood glucose levels. However, a substantial decline in the fasting blood glucose levels was observed following the administration of the methanolic extract of *S*. *filicaulis* at 150 mg/kg and 300 mg/kg (Table 2). Both doses yielded similar results to the standard drug metformin (at 100 mg/kg). The blood glucose levels of the normal control group remained stable throughout the experiment.

**Table 2:** Fasting blood glucose levels in fructose-fed streptozotocin-induced diabetic rats.

Animals	Initial (mg/dl)	72 h (mg/dl)	15 days (mg/dl)
Control	81.33 ± 4.93	83.33 ± 4.16	86.33 ± 7.57
STZ control	86.01 ± 2.00	436.24 ± 35.54	400.21 ± 29.62
STZ + metformin (100 mg/kg)	79.66 ± 6.43*^#^	501.02 ± 14.73*^#^	92.16 ± 12.17*^#^
STZ + MESFL (150 mg/kg)	83.66 ± 8.39*^#^	483.66 ± 20.21*^#^	95.33 ± 6.43*^#^
STZ + MESFL (300 mg/kg)	89.33 ± 8.61*^#^	400.33 ± 36.55*^#^	88.37 ± 10.54*^#^

Data are presented as the mean ± SEM of five rats in each group.

* Indicates significant differences from the diabetic control group (P value < 0.05);

^#^ signifies significant differences from control animals.

Legends: STZ: streptozotocin; MESFL: Methanolic extract of *Spilanthes filicaulis* leaves

#### HOMA-IR and HOMA- β levels of diabetic rats administered MESFL

Table 3 shows the serum insulin, HOMA-IR, and HOMA-β scores in the STZ-induced diabetic rats. Serum insulin levels was significantly reduced in diabetic rats whereas metformin and MESFL administration increased the levels. HOMA-IR scores increased after the induction of T2D, in contrast, treatment with metformin and MESFL reduced the scores in all the treatment groups. On the contrary, the HOMA-β score was reduced in diabetic rats whereas MESFL and metformin increased the scores in the treated groups, which all compared well to the normal control group.

**Table 3:** HOMA-IR and HOMA-β scores of streptozotocin-induced diabetic rats after administration of methanolic extract of Spilanthes filicaulis leaves.

GROUPS	PARAMETERS		
	INSULIN (U/l)	HOMA-IR	HOMA-β
Control	15.41 ± 0.07^e^	7.02 ± 0.12^a^^b^	49.52 ± 1.24^b^^c^
STZ control	10.09 ± 0.56^a^	11.04 ± 0.55^c^	5.07 ± 0.02^a^
STZ + metformin (100 mg/kg)	13.23 ± 1.01^b^	6.31 ± 0.70^a^	36.65 ± 1.35^b^
STZ + MESFL (150 mg/kg)	14.05 ± 1.04^c^	6.40 ± 0.51^a^	58.02 ± 1.46^c^
STZ + MESFL (300 mg/kg)	14.47 ± 0.57^c^^d^	8.56 ± 0.71^a^^b^	38.12 ± 1.22^b^^c^

Data are expressed as mean ± SD (n = 5).

^a-e^Values with different letters along a column for a given parameter are significantly different (P <0.05) from each other.

* MESFL: Methanolic Extract of *Spilanthes filicaulis* leaves.

*HOMA-IR (Homeostatic model assessment of insulin resistance): [(Fasting serum insulin in U/l *fasting blood glucose in mmol/l)/22.5]

*HOMA-β (Homeostatic model assessment of β-cell function: [(Fasting serum insulin in U/l *20/fasting blood glucose in mmol/l-3.5)]

*Conversion factor: Insulin (1U/l = 7.174pmol/l)

#### Renal antioxidant activities of streptozotocin-induced diabetic rats administered a methanolic extract of *Spilanthes filicaulis* leaves

As presented in Table 4, catalase and superoxide dismutase (SOD) activities and reduced glutathione (GSH) levels in the diabetic control group were significantly lower. However, the administration of metformin as well as MESFL at doses of 150 mg/kg and 300 mg/kg resulted in a significant increase in catalase and SOD activities and GSH levels. In contrast, the levels of MDA in the diabetic group were higher than those in all other groups. A decrease in MDA levels was observed in diabetic rats treated with the methanolic extract of *Spilanthes*, *filicaulis* and metformin.

**Table 4:** Renal antioxidant marker of streptozotocin-induced Wistar rats after oral administration of methanolic extract of *Spilanthes filicaulis* leaves.

PARAMETERS	GROUPS				
	Normal Control	Diabetic Control	Diabetes + Metformin	Diabetes + MESFL (150 mg/kg)	Diabetes + MESFL (300 mg/kg)
**Catalase** (mmol/min/mg protein)	34.36±3.13^b^	15.32±0.44^a^	25.54±5.54^b^^,^^c^	26.79±3.15^b^^,^^c^	30.79±1.04^b^
**GSH** (μmol/mL)	59.65±2.85^b^	45.50±0.83^a^	75.09±11.12^d^	89.98±6.71^e^	63.60±0.37^b^^,^^c^
**SOD** (mmol/min/mg protein)	2.64±0.22^b^	1.22±0.04^a^	2.19±0.38^b^	2.30±0.25^b^	2.32±0.13^b^
**MDA** (μmol/mL)	1.69±0.25^b^	2.76±0.06^a^	1.67±0.59^b^	1.25±0.11^b^	1.21±0.71^b^

Data are expressed as the mean ± SD (n = 5).

^a-e^Values with different superscripts are significantly different along the row.

Legend: **MESFL**: Methanolic Extract of *Spilanthes filicaulis* leaves.

**MDA:** malondialdehyde, **SOD**: superoxide dismutase, **GSH**: reduced glutathione,

#### Selected renal and metabolic function markers

Table 5 shows the selected renal and metabolic function markers in diabetic rats. We noticed significant enhancement in the levels of ALT, AST, ALP, serum creatinine, urea, and uric acid in streptozotocin-induced diabetic rats. Administration of 150 and 300 mg/kg MESFL or metformin to diabetic rats revealed considerable reductions in these parameters (Table 5).

**Table 5:** Effect of the methanolic extract of *S*. *filicaulis* leaves on selected renal and metabolic function markers in streptozotocin-induced Wistar rats.

Parameters	GROUPS				
	Normal Control	Diabetic Control	Diabetic +Metformin	Diabetic +MESFL (150 mg/kg)	Diabetic +MESFL (300 mg/kg)
Serum ALP (U/L)	14.30±1.59^a^	28.90±1.18^b^	19.30±3.27^a^^,^^c^	9.57±3.11^d^	17.44±0.10^a^^,^^c^
Kidney ALP (U/L)	23.15±3.90^a^	51.36±0.32^b^	21.11±4.45^a^	20.61±2.22^a^	17.78±2.78^a^^,^^c^
Serum ALT (U/L)	21.66±2.31^a^	54.01±2.22^b^	35.19±5.51^c^	26.23±1.61^a^	32.38±0.94^c^
Serum AST (U/L)	40.29±3.96^a^	51.17±1.92^b^	40.63±10.01^a^	25.60±2.65^d^	36.83±3.92^a^^,^^c^
Serum creatinine (mg/dL)	0.26±0.04^a^	2.29±0.10^b^	0.90±0.30^a^	1.10±0.20^c^	1.09±0.26^c^
Kidney Creatinine (mg/dL)	2.24±0.00^a^	7.46±0.00^b^	2.98±0.00^a^^,^^c^	2.98±0.75^a^^,^^c^	1.87±1.12^d^
Serum Urea (mg/dL)	130.57±9.33^a^	210.06±12.81^b^	133.42±3.99^a^	127.33±9.54^a^	118.01±6.24^a^^,^^c^
Kidney Urea (mg/dL)	122.38±0.00^a^	278.29±19.77^b^	136.07±1.52^c^	128.47±12.17^a^	126.93±9.12^a^
Serum Uric Acid (mg/dL)	3.62±0.31^a^	9.73±0.26^b^	3.76±0.69^a^	4.02±0.46^c^	3.08±0.09^a^
Kidney Uric Acid (mg/dL)	9.78±0.83^a^	18.74±0.23^b^	11.15±1.19^c^	12.05±0.44^d^	10.81±0.02^a^^,^^c^

Data are presented as the mean ± SEM (n = 5).

^a-d^Values with different letters along a given row for a given parameter are significantly different (Duncan multiple range post hoc, p<0.05) from each other.

*MESFL: Methanolic extract of *S*. *filicaulis* *ALP: Alkaline Phosphatase

*ALT: Alanine aminotransaminase *AST: Aspartate aminotransaminase

#### The cAMP/PKA/CREB/cFOS pathway was suppressed in the renal tissues of diabetic rats

**Fig 1:**
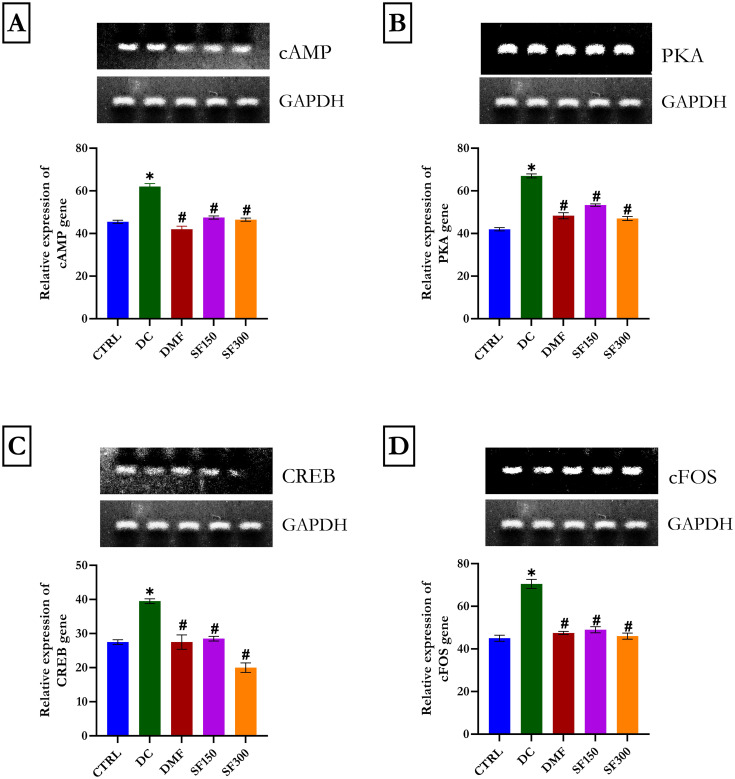
cAMP, PKA, CREB, and c-Fos gene expression in rats in each group. The expression of cAMP (Fig 1), PKA (Fig 1B), CREB (Fig 1C), and c-Fos (Fig 1D) was analyzed by RT–qPCR. Data are presented as the mean ± SEM (n = 5). *p < 0.05 compared with the normal control group. #p < 0.05 compared with the diabetic control group. GAPDH was used as a loading protein.

As revealed in Fig 1A–1D, cAMP, PKA, CREB, and cFOS levels were significantly elevated in the renal tissues of diabetic rats using RT–PCR. Administration of 150 and 300 mg/kg MESFL significantly downregulated these effects. These results compared well with those of the metformin-treated group.

#### Effect of *Spilanthes filicaulis* methanolic extract on Bcl-2

**Fig 2:**
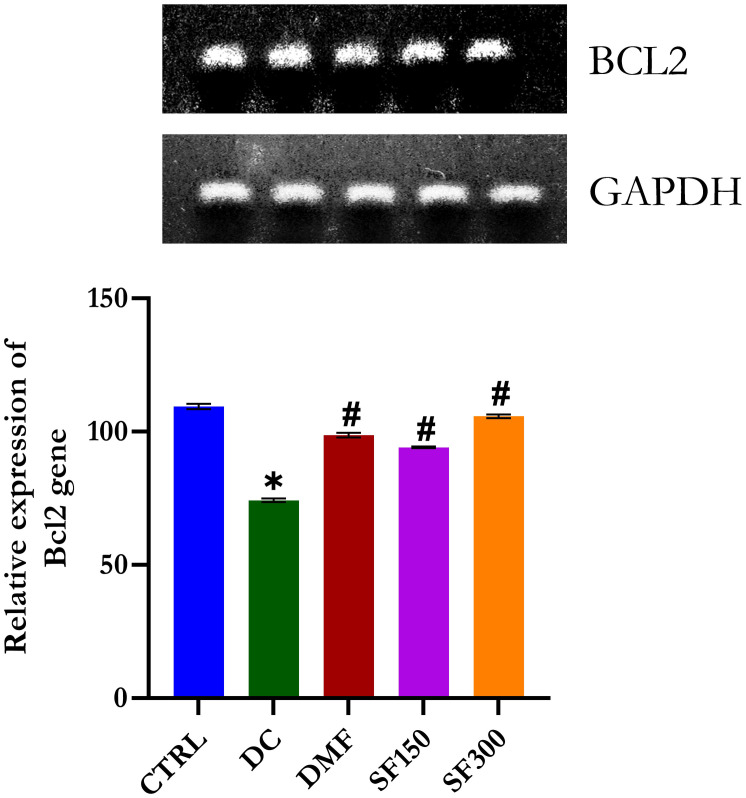
RT–PCR analysis of Bcl2 in the kidneys of diabetic rats. Data are presented as the mean ± SEM (n = 5). *p < 0.05 compared with the normal control group. #p < 0.05 compared with the diabetic control group. GAPDH was used as a loading protein.

Fig 2 shows that Bcl2 exhibited a significant (p < 0.05) reduction in the kidneys of diabetic rats, which indicated apoptotic cell damage in kidney tissues with the progression of DM. Treatment with metformin and MESFL, however, could significantly (p < 0.05) upregulate the less-expressed Bcl2 in the kidney of diabetic rats.

### Histopathology of the kidney tissues

**Fig 3:**
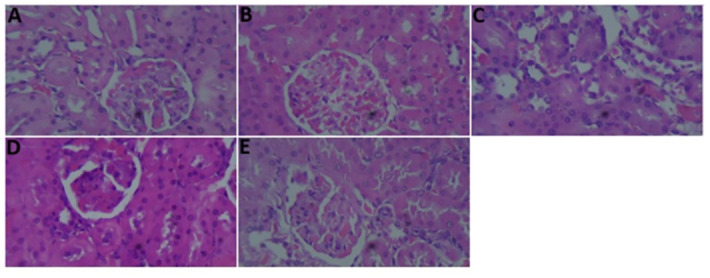
Histological micrographs showing the effect of *Spilanthes filicaulis* and metformin in streptozotocin-induced diabetic rat kidney tissues using a hematoxylin and eosin staining technique, A (Control), B (Diabetic Control), C (Metformin) D (MESFL 150 mg/kg) and E (MESFL 300 mg/kg). **Scale bar**: 50 μm, **magnification**: 400x.

As shown in Fig 3A, there was a normal histoarchitecture of the nephrocytes in the control rats, while in the STZ-induced diabetic rats, the kidney tissue showed a thickened basement membrane and degenerated glomerulus (Fig 3B). Administration of the methanolic extract of *Spilanthes filicaulis* or metformin preserved the basement membrane and degenerated glomerulus (Fig 3C–3E).

## Discussion

Diabetes mellitus (DM) is often associated with a fatal condition called diabetic nephropathy, which is the most common reason for terminal-stage renal failure. Approximately 15% to 25% of type 1 diabetic patients and 30% to 40% of type 2 diabetic patients with DM have diabetes nephropathy [43]. Type 2 diabetes mellitus (T2DM) disrupts various metabolic processes in the body, leading to a wide range of symptoms [44]. The management of T2DM focuses on alleviating these symptoms, particularly hyperglycemia [45].

In this study, we administered a combination of fructose and low-dose streptozotocin to experimental rats. The combination led to hyperglycemia with glycemic imbalance, renal dysfunction, and apoptosis. According to Wilson and Islam [32], fructose and streptozotocin-induced type 2 diabetes serves as an effective experimental paradigm for examining the antidiabetic effects of various drugs. Fructose and streptozotocin cause T2DM by selectively destroying pancreatic β-cells [32]. We observed an increase in fasting blood glucose (FBG) levels after administration of low-dose streptozotocin. This may be attributed to the selective cytotoxicity of STZ on β-cells [32]. The decrease in serum FBG levels observed in the MESFL-treated and metformin-treated groups compared to the control group supports the idea that the medicinal properties of MESFL may be mediated by secondary metabolites [46].

T2DM is as a results of insulin resistance and partial destruction of pancreatic β-cells [46, 47]. The administration of MESFL lowered the blood glucose while also elevating insulin levels in the serum. The improvement in insulin secretion by MESFL results in a reduction in blood glucose level, as other studies have documented various natural products produce their normoglycaemic effect [48]. The diabetic control group had elevated HOMA-IR and reduced HOMA-β scores, indicating partial pancreatic β-cell dysfunction and insulin resistance, as previously documented [46, 47]. MESFL ability to reverse HOMA-IR and HOMA-β scores suggests reduced insulin resistance and regeneration of β-cells.

Recent studies have shown that the buildup of reactive oxygen species (ROS) in hyperglycemic circumstances can lead to kidney damage [49, 50]. In particular, Kiritoshi et al. [51] discovered a significant increase in the number of ROS in cells in the glomerulus. Alterations in MDA and enzymatic antioxidants reveal oxidative stress in these cells. Furthermore, lipid peroxidation increases the risk of oxidative stress, which may lead to a reduction in enzymatic antioxidants. In streptozotocin-induced diabetic nephropathy, superoxide dismutase (SOD), catalases (CAT), and glutathione reduced (GSH) have been reported to be involved in previous studies [49, 52]. MESFL or metformin treatment in diabetic rats significantly inhibited lipid peroxidation and increased GSH, SOD, and CAT. These findings are in line with the report of [49].

In this study, damage to renal function was observed in diabetic rats induced by STZ, as indicated by significant elevations in ALT, AST, and ALT, as well as urea, uric acid, and creatinine. These findings are in line with those observed in previous studies [49, 53, 54]. Treatment with *Spilanthes filicaulis* (150 and 300 mg/kg bw/t) significantly reduced the progression of diabetes nephropathy, as evidenced by a significant decrease in renal function markers (ALP, uric acid, urea and creatinine).

The cAMP/PKA/CREB/c-FOS signaling pathway is one of the classical pathways presently being studied. cAMP is a second messenger molecule. Levels of cAMP are affected by cAMP-dependent protein kinase (PKA). PKA is composed of two regulatory subunits plus two catalytic subunits. When cAMP binds to the regulatory subunits of PKA, it initiates a conformational shift that allows the catalytic subunits to be released and activated. This changes the target protein by controlling cAMP response element-binding protein (CREB). The CREB protein then phosphorylates the appropriate target protein molecules in the cell and regulates gene transcription, resulting in the biological effects of the cAMP/PKA/CREB/c-FOS signaling pathway [55–57]. In response to a variety of extracellular signals, cells generate cyclic AMP (cAMP) by the catalytic dephosphorylation of ATP (adenosine triphosphate) through the action of adenylyl cyclase enzymes. Studies have shown that the primary mechanisms of adenylyl cyclase activation are the activation of G protein-coupled receptor (GPCR) and β-adrenoreceptor (β-ADR) receptors, as well as glucagon receptors (GCGR) to heterotrimeric G-proteins. Cyclic AMP (cAMP)-dependent protein kinase A (PKA) regulates a wide range of cellular functions by reversibly phosphorylating key substrates [58, 59]. All of these pathways are related to PKA, the regulatory kinase. cAMP controls gene expression by mediating the phosphorylation of the CREB transcription factor at Ser-133, which is dependent on PKA. CREB, a substrate of PKA, is phosphorylated and interacts with CRE in the nucleus to control gene expression and protein synthesis [60]. In the kidney tissue of diabetic rats treated with MESFL or metformin, cAMp/PKA/CREb/cFOS was reduced significantly. These findings suggest a relationship between cAMP/PKA/CREB/cFOS signaling and diabetic nephropathy. The results of this study revealed that MESFL downregulates the cAMP/PKA/CREB/cFOS signaling pathway in kidney cells.

Bcl-2 is a protein that plays a critical role in the regulation of cell apoptosis, and upregulation of its expression promotes cell survival [49, 61–64]. In this study, the downregulation of renal expression of Bcl-2 in STZ-induced diabetic rats induced diabetic nephropathy. However, in diabetic rats treated with *Spilanthes filicaulis* or metformin, the expression of this protein was increased, encouraging cell survival. The results of this study are similar to those of previous studies [33, 49, 54].

In this study, the histology of the kidney was investigated, and light microscopy following the H and E technique revealed that the control rats that received distilled water presented a normal histomorphology and that the nephrocytes were intact. In contrast, the kidney tissue was affected in the STZ-induced rats, showing a thickened basement membrane and degenerated glomerulus [65]. However, in the metformin and methanolic extracts of *Spilanthes filicaulis*-treated rats, these features were preserved.

## Conclusion

This study shows that *Spilanthes filicaulis* alleviates oxidative stress and the development of diabetic nephropathy via downregulation of the cAMP/PKA/CREB/cFOS signaling pathway and upregulation of the antiapoptotic protein Bcl-2. These findings suggest that the protective ability of *S*. *filicaulis* could be a result of antioxidant and renoprotective activities. Through cAMP/PKA/CREB/cFOS signaling pathways, *Spilanthes filicaulis* may slow the progression of diabetic nephropathy in rats. This study has limitation in that there are some parameters such as level of urine was not detected.

## Supporting information

S1 Data (ZIP)

S1 Raw images (ZIP)
