# Supplementary figures and images for: Spilanthes filicaulis (Schumach. & Thonn.) C.D. Adams leaves protects against streptozotocin-induced diabetic nephropathy
Source: PLoS One. 2024 Apr 19;19(4):e0301992. doi: 10.1371/journal.pone.0301992 (PMC11029641; doi:10.1371/journal.pone.0301992)

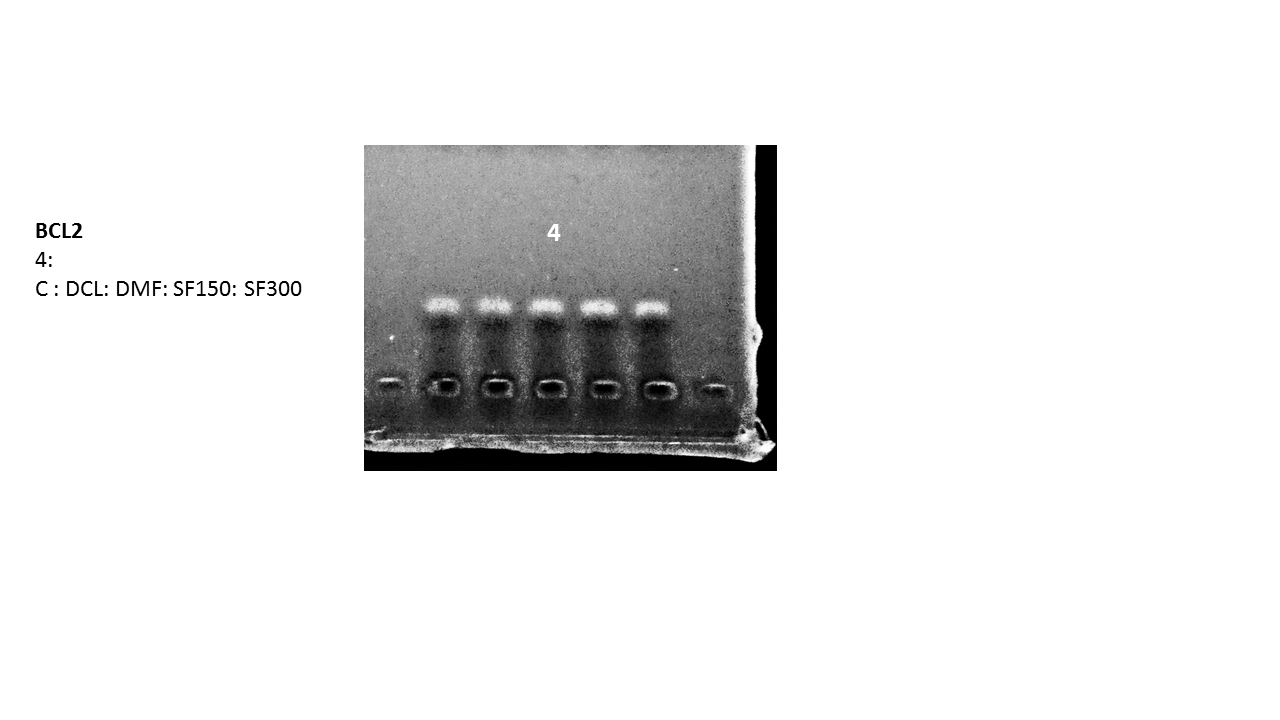

Supplement: S1 Raw images — (ZIP) [file pone.0301992.s002.zip › BCL2-Kid.TIF]

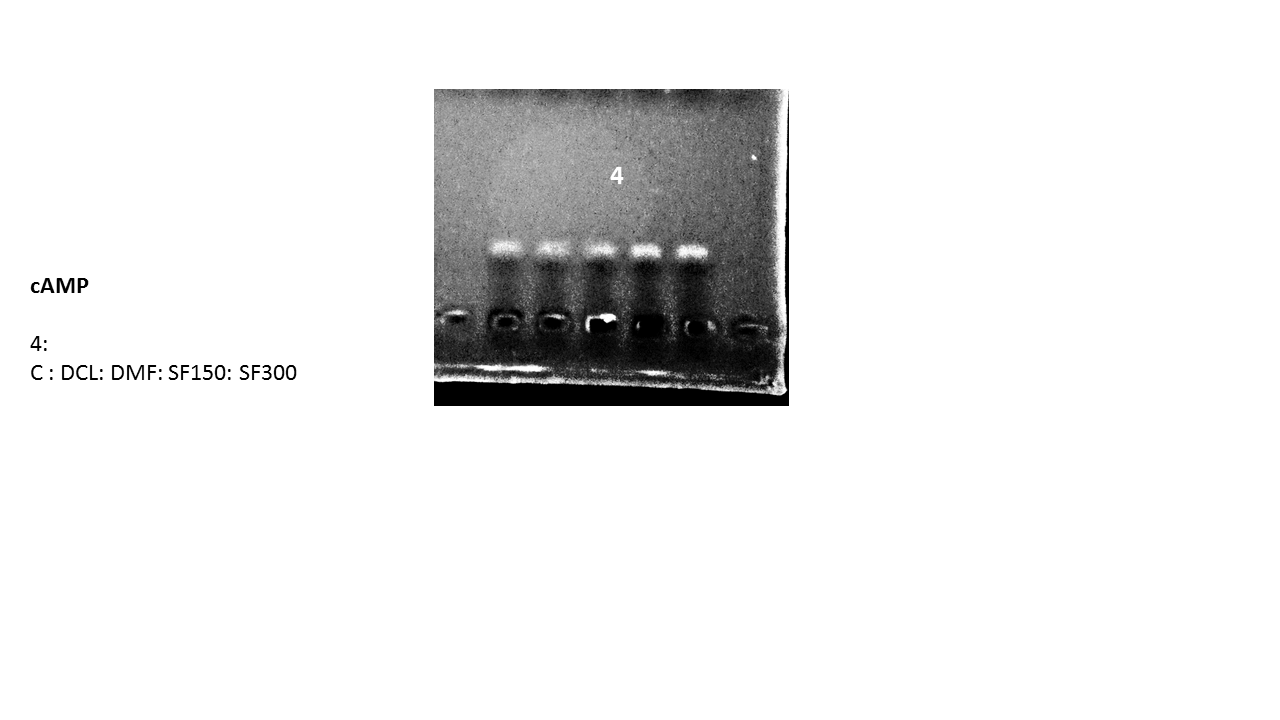

Supplement: S1 Raw images — (ZIP) [file pone.0301992.s002.zip › cAMP_Kid.TIF]

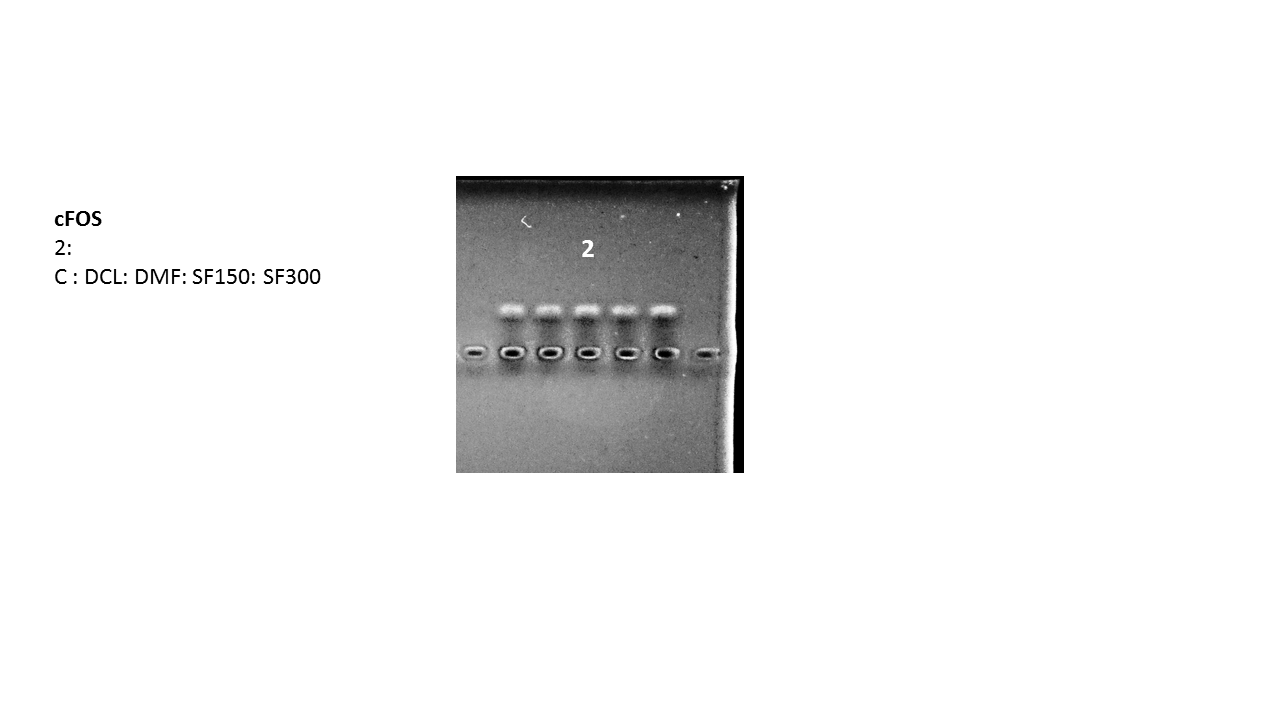

Supplement: S1 Raw images — (ZIP) [file pone.0301992.s002.zip › cFOS-Kid.TIF]

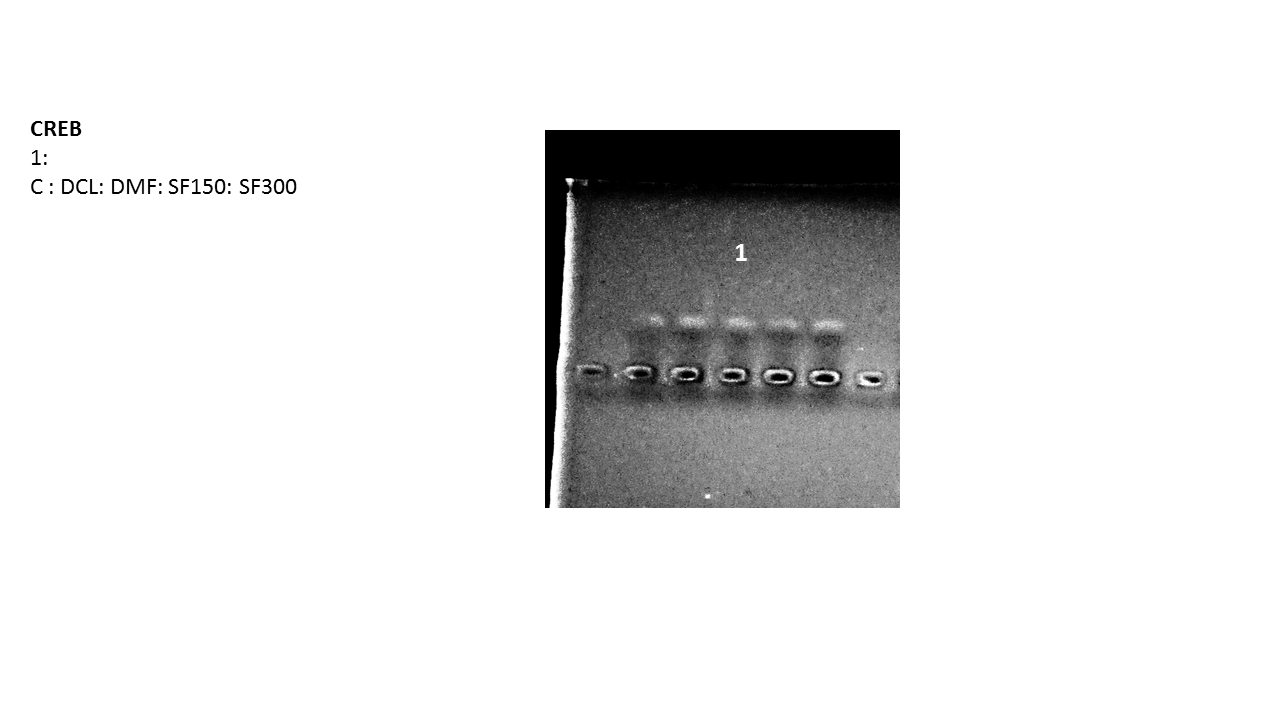

Supplement: S1 Raw images — (ZIP) [file pone.0301992.s002.zip › CREB-Kid.TIF]

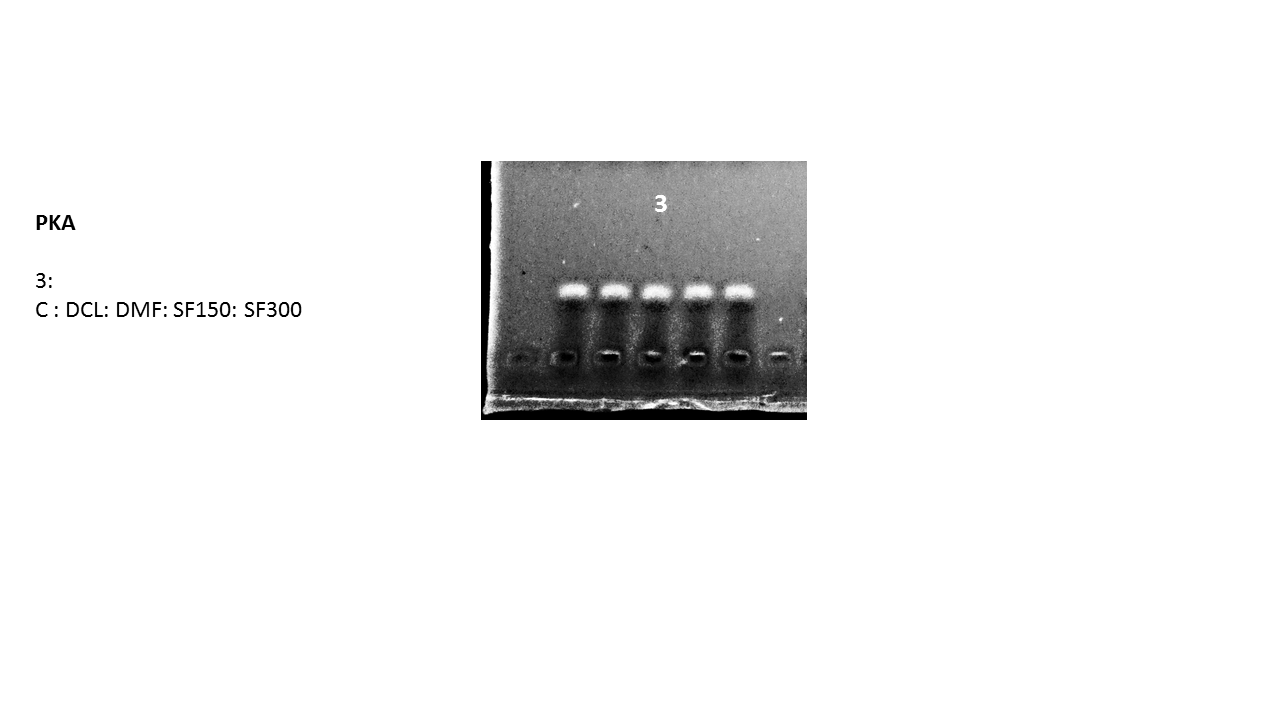

Supplement: S1 Raw images — (ZIP) [file pone.0301992.s002.zip › PKA_Kid.TIF]
